# Temporal Anti-Parity–Time Symmetry: Extending Non-Hermitian Physics to Time-Domain Dynamics in Thermal Diffusion

**DOI:** 10.34133/research.1187

**Published:** 2026-03-24

**Authors:** Haoran Yan, Ying Li

**Affiliations:** ^1^ State Key Laboratory of Extreme Photonics and Instrumentation, Key Lab. of Advanced Micro/Nano Electronic Devices & Smart Systems of Zhejiang, Zhejiang University, Hangzhou 310027, China.; ^2^ International Joint Innovation Center, The Electromagnetics Academy at Zhejiang University, Zhejiang University, Haining 314400, China.; ^3^ Shaoxing Institute of Zhejiang University, Zhejiang University, Shaoxing 312000, China.

## Abstract

Parity–time and anti-parity–time symmetries, originally formulated in the context of non-Hermitian quantum mechanics, have now been experimentally realized across diverse physical platforms, including photonic, wave-guiding, acoustic, and mechanical systems, as well as systems operating near exceptional points. While the active manipulation of these symmetries has been well explored in wave-based systems, their implementations in diffusive physics have long remained predominantly confined to static phases, where systems are locked in a fixed symmetric or symmetry-broken configuration due to their invariant structural parameters. Bridging this gap, recent work has introduced and verified the concept of temporal anti-parity–time symmetry, demonstrating that energy transport in diffusive media can be actively programmed in the time domain.

Anti-parity–time (APT) symmetry, which is characterized by the transformation of the system’s Hamiltonian into its negative (H→−H) by parity and time-reversal operators, often leads to purely imaginary eigenvalues and exhibits fruitful noteworthy phenomena; together with parity–time (PT) symmetry, it plays a critical role in describing non-Hermitian systems across diverse physical platforms, ranging from electronics [1,2], optics [3,4], acoustics [5,6], and time-varying systems [7] to thermodynamics [8–10]. While the active manipulation of APT symmetry and its singularities has been demonstrated [4,11,12], research on PT and APT symmetries within non-Hermitian diffusive systems has long remained tethered to static regimes, where systems are typically locked in invariant configurations (see Fig. 1A). Early studies, such as those by Xu et al. [8], have achieved control of parameters in thermal metamaterials through rotating modules; however, extending symmetry protection from the parameter space to the pure time domain remains a challenge. Meanwhile, diffusion systems (heat conduction and mass transfer) have long been considered incapable of representing similar symmetry dynamics due to the absence of oscillation or the coherent state in diffusion. Writing in *Nature Physics*, Jiping Huang’s group at Fudan University, together with Cheng-Wei Qiu’s group at the National University of Singapore, has recently reported temporal APT symmetry in a 3-ring thermal system with time-tailored lattice coupling, accomplishing substantial expansion from traditional non-Hermitian physics to the time domain (see the schematic diagram in Fig. 1B) [13].

**Fig. 1.**
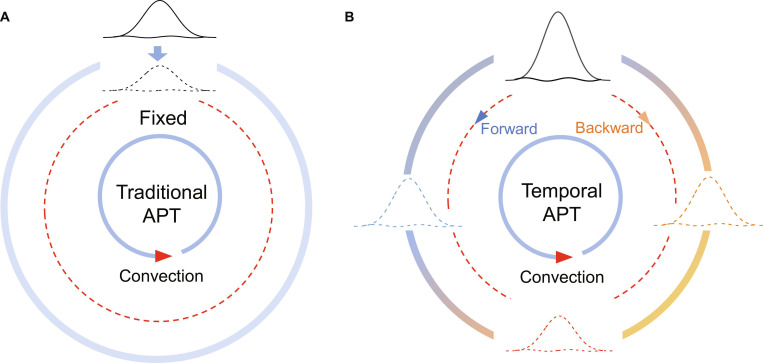
The schematic diagrams of traditional anti-parity–time (APT) symmetry and temporal APT symmetry. (A) Traditional APT symmetry traps a thermal wave packet within a convective medium (arrows indicate the flow direction). As time progresses, the wave packet remains localized but gradually dissipates, shown as a fainter packet at the same position. (B) Temporal APT symmetry, under identical convective conditions, allows the active trapping of heat at arbitrary locations. By implementing temporal modulation, heat transport (with accompanying dissipation) can occur either forward (aligned with convection) or backward (against convection), enabling tunable spatial control.

In this work, Jin et al. implement temporal APT symmetry in a 3-ring thermal metadevice, where programmable convection from 2 counter-rotating outer rings and a dynamically reconfigurable central ring jointly realize time-tailored thermal coupling, thereby establishing a temporally balanced gain–loss platform. The authors focus particularly on thermal trapping to demonstrate dynamic energy manipulation. By simultaneously tuning the material composition of the central ring and resetting the rotational speeds of the outer rings, they impose square-wave modulation on the angular velocities. A designated switching time *t*_0_ marks the moment when both the ring rotation and the heat exchange rate *h* between the inner and outer rings transition from *h* < *h*^EP^ to *h* > *h*^EP^, where *h*^EP^ denotes the exceptional-point threshold. Before *t*_0_, the system behaves as if driving a thermal wave packet; after *t*_0_, it transitions into a “trapping phase”.

The experimental realization employs an apparatus composed of a time-varying metamaterial, a motor-driving rotation module, an electronic control module, and a pneumatic actuator that switches embedded materials between air and copper in the aluminum alloy ring at *t*_0_; there is a hot-air gun used to continuously heat a point for 45 s. Temperature measurements at locations one-third and two-thirds cycle away from the heating position confirm the occurrence of thermal trapping at the expected position, consistent with theoretical predictions.

Determining the precise *t*_0_ is crucial for achieving dynamic control of the APT symmetry of the system and accurate regulation of heat transfer. However, because the thickness of the central ring cannot be ignored, it affects the heat transfer of the entire structure to a certain extent; a normal analytical method makes it difficult to predict the thermal evolution of this system, and a deep-learning-model-based artificial neural network (ANNs) is developed to manufacture a metamaterial with an adjustable *t*_0_ and determine the optimal *t*_0_, committed to driving the wave packet arriving at the target location *x*_T_ from the initial location *x*_0_. This ANN-based strategy marks a major step forward in the control and manipulation of thermal systems, providing a powerful means to design metamaterials with tunable thermal responses.

Collectively, this work introduces the concept of temporal APT symmetry, which extends APT physics into the time domain. The authors verify this concept experimentally and demonstrate programmable control over thermal energy transport. Their 3-ring platform offers a new route for exploring time-dependent non-Hermitian physics beyond static phase classification. In addition, the deep-learning strategy based on ANNs provides a practical and accurate approach for designing thermal metamaterials with tunable temperature wave packets and robust performance in complex systems.

In summary, the transition from static to temporal APT symmetry marks a critical shift in non-Hermitian diffusive physics. However, several concrete challenges must be addressed to fully unlock its potential. First, the physical limits of switching speed in temporal programming—governed by the intrinsic thermal relaxation time—remain to be quantified. Second, while this 3-ring platform demonstrates fundamental principles, scaling such architecture to complex, multinode diffusive networks presents important engineering hurdles. Future research should focus on practical application scenarios, such as dynamic thermal routing in high-performance microprocessors or adaptive energy management in thermoelectric systems, where active temporal control could offer efficiency gains over passive designs. Furthermore, while this approach hints at a broader framework for energy control, its extension to quantum systems must be treated with caution. In open quantum systems, effective non-Hermitian descriptions are valid only when quantum jump terms in the Lindblad master equation are negligible. Bridging this classical temporal engineering with quantum frontiers, such as observing quantum temporal correlations in PT-symmetric qubits [14], may eventually yield a more nuanced and rigorous unified theory for information and energy exchange across physical scales.
